# Transcriptome Sequencing of *Dianthus spiculifolius* and Analysis of the Genes Involved in Responses to Combined Cold and Drought Stress

**DOI:** 10.3390/ijms18040849

**Published:** 2017-04-17

**Authors:** Aimin Zhou, Hongping Ma, Enhui Liu, Tongtong Jiang, Shuang Feng, Shufang Gong, Jingang Wang

**Affiliations:** 1 College of Horticulture and Landscape Architecture, Northeast Agricultural University, Harbin 150030, China; aiminzhou@neau.edu.cn (A.Z.); hongpingma0818@163.com (H.M.); liuenhui1024@163.com (E.L.); tongtongjiang7@163.com (T.J.); 2 Key Laboratory of Saline-Alkali Vegetation Ecology Restoration in Oil Field (SAVER), Ministry of Education, Alkali Soil Natural Environmental Science Center (ASNESC), Northeast Forestry University, Harbin 150040, China; fengshuang86@163.com

**Keywords:** *Dianthus spiculifolius*, cold, drought, combined stress, transcriptome, differentially expressed genes

## Abstract

*Dianthus spiculifolius*, a perennial herbaceous flower and a member of the Caryophyllaceae family, has strong resistance to cold and drought stresses. To explore the transcriptional responses of *D. spiculifolius* to individual and combined stresses, we performed transcriptome sequencing of seedlings under normal conditions or subjected to cold treatment (CT), simulated drought treatment (DT), or their combination (CTDT). After de novo assembly of the obtained reads, 112,015 unigenes were generated. Analysis of differentially expressed genes (DEGs) showed that 2026, 940, and 2346 genes were up-regulated and 1468, 707, and 1759 were down-regulated in CT, DT, and CTDT samples, respectively. Among all the DEGs, 182 up-regulated and 116 down-regulated genes were identified in all the treatment groups. Analysis of metabolic pathways and regulatory networks associated with the DEGs revealed overlaps and cross-talk between cold and drought stress response pathways. The expression profiles of the selected DEGs in CT, DT, and CTDT samples were characterized and confirmed by quantitative RT-PCR. These DEGs and metabolic pathways may play important roles in the response of *D. spiculifolius* to the combined stress. Functional characterization of these genes and pathways will provide new targets for enhancement of plant stress tolerance through genetic manipulation.

## 1. Introduction

Adverse environmental conditions seriously affect the growth and development of plants. In particular, abiotic stresses such as cold, heat, drought and salinity have large detrimental effects on agricultural production. Investigations into the effects of stress have generally examined the responses to individual abiotic stresses [1,2]; however, abiotic stresses rarely occur individually and plants are often simultaneously exposed to a combination of stresses such as cold and drought. In the field, the combination of cold and drought is recognized as an important threat to plants during early spring and winter in temperate regions [3]. To date, the molecular mechanisms associated with plant responses to combined cold and drought stresses, such as changes in gene expression, signal transduction and regulatory networks, are largely unknown.

Transcriptome profiling has been carried out in various plant species to characterize their responses to individual stresses [4,5,6,7,8,9,10]. Only a few studies have investigated the molecular mechanisms underlying the tolerance to combined cold and drought stress. In *Arabidopsis*, plants with mutation in *ein*2 (ethylene insensitive) and *aba*1.6 (abscisic acid) show differential regulation of 2313 and 4131 transcripts, respectively, under cold and drought stress. Validation of some of the important differentially expressed genes (DEGs) showed that *HSPs* (heat shock proteins), *COR*47 (cold regulated proteins), and *SPX*1 (SPX domain containing proteins) were altered by stress in *ein*2 and *aba*1.6 mutants [11]. Furthermore, overexpression of an exogenous *HSP* and *COR* increases multiple abiotic stress tolerance in transgenic plants [12,13]. In tea (*Camellia sinensis*), 319 DEGs that enhance the resistance to combined cold and drought stress have been identified. Additionally, drought-induced leaf senescence is affected by the expression of senescence-associated genes and cold responsive genes and by the regulation of multiple metabolic pathways in response to a combined stress [14].

In contrast to model and economically important species, many wild plant species are able to grow in stressful environments because of their specialized genetic resources and adaptive mechanisms. *Dianthus spiculifolius* Schur, a perennial herbaceous flower and a member of Caryophyllaceae [15], shows strong resistance to cold and drought stresses and can survive winters in Russian Siberia and northeast China, where temperatures can fall to −40 °C. This strong adaptability suggests that *D. spiculifolius* may have distinct molecular mechanisms that allow it to tolerate extreme conditions. However, to date, no genomic information has been reported for *D. spiculifolius*.

In the present study, we sought to elucidate the adaptation mechanisms of *D. spiculifolius* to complex environmental conditions by examining the transcriptome profiles of seedlings grown under either cold or drought stress conditions, as well as a combination of the two stresses. We identified DEGs in cold-treated (CT), simulated drought-treated (DT), and combined cold and simulated drought-treated (CTDT) plants, and also identified numerous specific stress-related genes. We also analyzed the signal transduction and metabolic pathways associated with these DEGs. Our results provide a basis for understanding the mechanisms of the responses of *D. spiculifolius* to the combined stress and also provided a potentially valuable resource for the future development of stress-tolerant plants through genetic manipulation.

## 2. Results and Discussion

### 2.1. Transcriptome Sequencing of D. spiculifolius Subjected to Cold, Drought, and Combined Cold and Drought Stress

**Figure 1:**
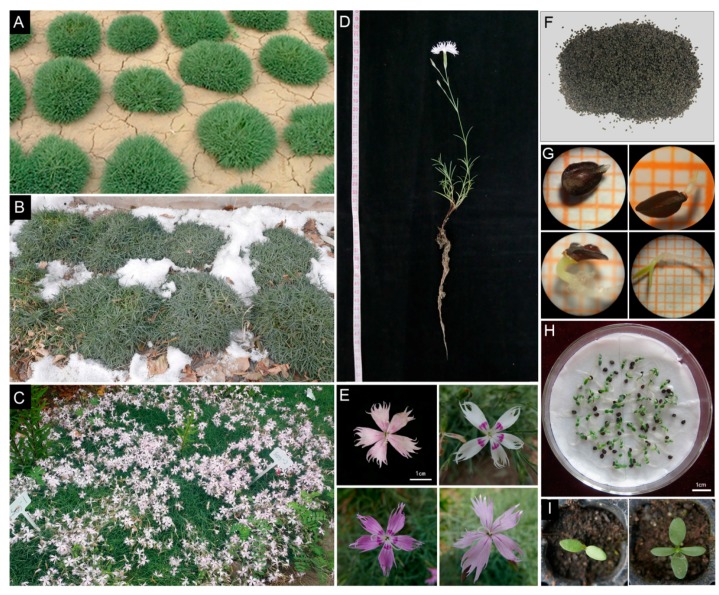
Open field cultivation and phenotypic characteristics of *D. spiculifolius*. (**A**–**C**) Open field cultivation of *D. spiculifolius* in a dry summer (**A**), cold winter (**B**) and autumn (**C**); (**D**,**E**) Phenotype of *D. spiculifolius* (**D**) and its flower type (**E**). (**F**) Seeds of *D. spiculifolius*; (**G**–**I**) Seed germination (**G**) and seedling growth (**H**,**I**) of *D. spiculifolius*.

*D. spiculifolius* plants cultivated in an open field showed strong resistance to cold and drought stresses (Figure 1A,B). The plants grew to 8–10 cm in height and flowered in the autumn (Figure 1C), producing needle-like leaves and flowers, 2 cm in diameter (Figure 1D,E). In addition to asexual reproduction, *D. spiculifolius* can be propagated by seeds (Figure 1F–I). To investigate the changes in the patterns of transcription in response to combined cold and drought stress, we exposed 10-day-old seedlings to cold, simulated drought, and combined cold and simulated drought stress for 24 h. The Illumina HiSeq 2000 platform was used to analyze transcriptome sequences from the treated seedlings. In total, we obtained approximately 29–30 million raw reads for all the samples. After data cleaning and the removal of invalid reads, we obtained 23,624,696, 22,495,628, 23,317,141, and 22,965,345 clean reads for the control (CK), CT, DT, and CTDT samples, respectively. The reads had 46.45%, 46.32%, 46.62%, and 42.10% guanine and cytosine (GC) content in the CK, CT, DT, and CTDT samples, respectively. After clustering the high-quality reads, 184,782 transcripts and 112,015 unigenes were obtained for all the samples. The unigenes had an average length of 819 bp (Table 1).

To validate and annotate the assembled unigenes, sequence similarity searches were conducted using sequence- and domain-based alignments. In total, 36,142 (32.27% of all the unigenes), 29,149 (26.55%), 29,739 (26.54%), 25,073 (22.38%), and 10,845 (9.68%) unigenes were found in SwissProt, PFAM (Protein family), GO (Gene Ontology), KOG (euKaryotic Ortholog Groups), and KO (KEGG Ortholog) databases, respectively (Table 2).

### 2.2. Identification of Differentially Expressed Genes (DEGs) Responding to Combined Cold and Drought Stress

**Figure 2:**
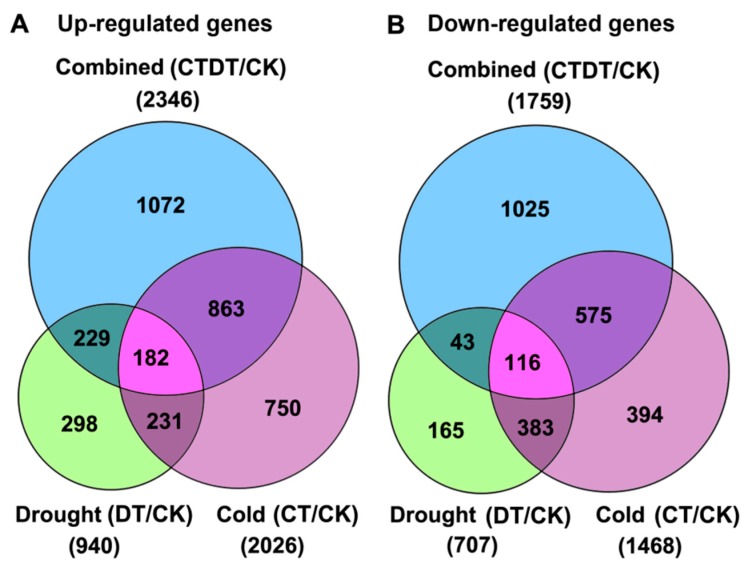
Venn diagrams showing the number of differentially expressed genes (DEGs). The numbers of up-regulated (**A**) and down-regulated (**B**) genes in either cold-, drought-, or combined cold and drought-treated samples of *D. spiculifolius* seedling (compared to the control non-stressed plants) are shown. We used “false discovery rate ≤0.05 and the absolute value of log_2_Ratio ≥1” as the threshold to judge the significance of the differences in gene expression.

To investigate the gene expression during cold and drought stress, we used the reads per kilobase per million reads (RPKM) method to calculate the expression levels of the unigenes from CT, DT, and CTDT samples compared to the CK sample. As shown in Figure 2, 940 up-regulated and 707 down-regulated unigenes were identified in the DT sample. In the CT sample, 2026 unigenes were up-regulated and 1468 were down-regulated. In the CTDT sample, 2346 unigenes were up-regulated and 1759 were down-regulated. Among all the DEGs, 182 up-regulated and 116 down-regulated overlapping DEGs were identified in all the three treatment groups, respectively (Figure 2, Tables S1 and S2). This suggested that these overlapping DEGs may play an important role in the response of plants to combined cold and drought stress.

### 2.3. Functional Classification and Expression Analysis of DEGs

**Figure 3:**
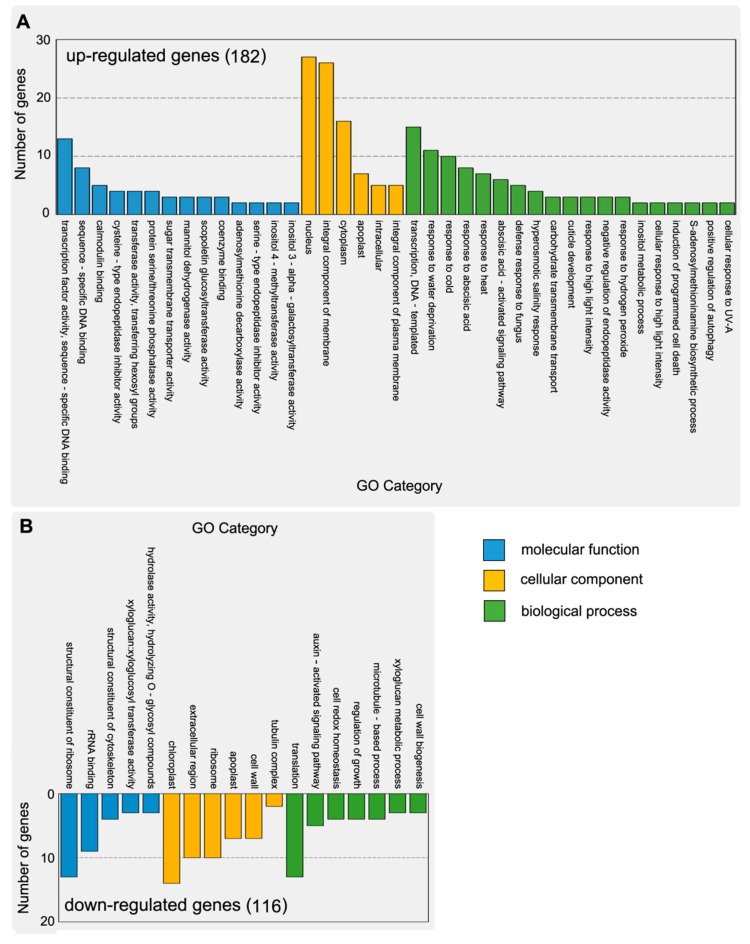
Histogram of gene ontology (GO) classification of DEGs. (**A**) GO classification of up-regulated genes. (**B**) GO classification of down-regulated genes. The results are summarized in three main categories: biological processes (green), cellular component (yellow) and molecular function (blue). The y-axis on the left side indicates the number of DEGs.

We used gene ontology (GO) annotation [16] to classify the possible functions of the overlapping DEGs. This analysis assigned the 182 up-regulated and 116 down-regulated DEGs to 39 and 18 functional groups, respectively; the three main categories were biological processes, cellular components, and molecular functions. In the molecular function category, the most frequent assignments of the up-regulated DEGs were transcription factor activity, sequence-specific DNA binding, and calcium binding. For cellular components, the DEGs were mostly assigned to nucleus, internal components of membrane, and cytoplasm. For biological processes, the majority of the DEGs were assigned to transcription, DNA-template, response to water dehydration, and cold (Figure 3A). The down-regulated DEGs were mainly assigned to the structural constituents of ribosome, chloroplast, and translation (Figure 3B).

**Figure 4:**
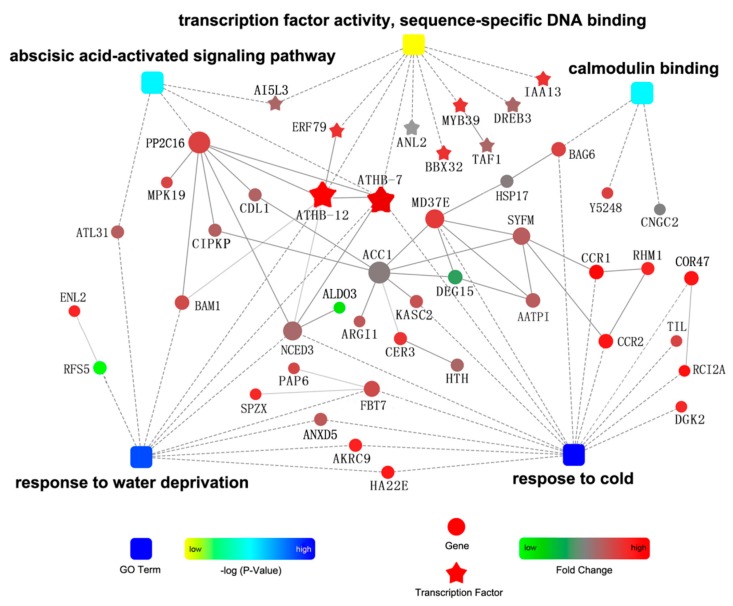
PPI sub-network involving the DEGs. The square nodes represent GO terms; circular nodes represent genes; star-like nodes represent transcription factors. Gray lines indicate interactions between two proteins. The intensity of the color of the nodes is based on the *p*-value of the GO term or fold change in the DEGs. All information for each gene can be found in Tables S1 and S2.

We analyzed the protein–protein interactions (PPI) of the DEGs involved in five important GO terms using STRING (http://string.embl.de/). A PPI subnetwork including 47 nodes and 36 interactions was constructed (Figure 4). The degree of connectivity of each node was calculated; the top six nodes were ACC1 (acetyl-CoA carboxylase 1), PP2C16 (type-2C protein phosphatase 16), ATHB7 and ATHB12 (homeobox-leucine zipper proteins 7 and 12), NCED3 (9-*cis*-epoxycarotenoid dioxygenase 3), and MD37E (mediator of RNA polymerase II transcription subunit 37e). The transcription factors ATHB7 and ATHB12 were considered as key nodes that interacted with many proteins, including the transcription factors ERF79 (ethylene response factor), PP2C16, and NCED3. It has been reported that ATHB7 and ATHB12 modulate abscisic acid (ABA) signaling by regulating PP2C [17]. The ABA signaling pathway is one of the important signaling pathways in plants for response to cold and drought stresses [18,19]. Furthermore, PP2C16 interacted with the protein kinases MPK19 (mitogen-activated protein kinase 19), CIPKP (calcineurin B-like protein (CBL)-interacting serine/threonine-protein kinase 25), and CDL1 (serine/threonine-protein kinase CDL1), which can catalyze the phosphorylation of proteins and are an important part of cell signal recognition and transduction in the responses of plants to abiotic stress [18,20,21]. 

**Figure 5:**
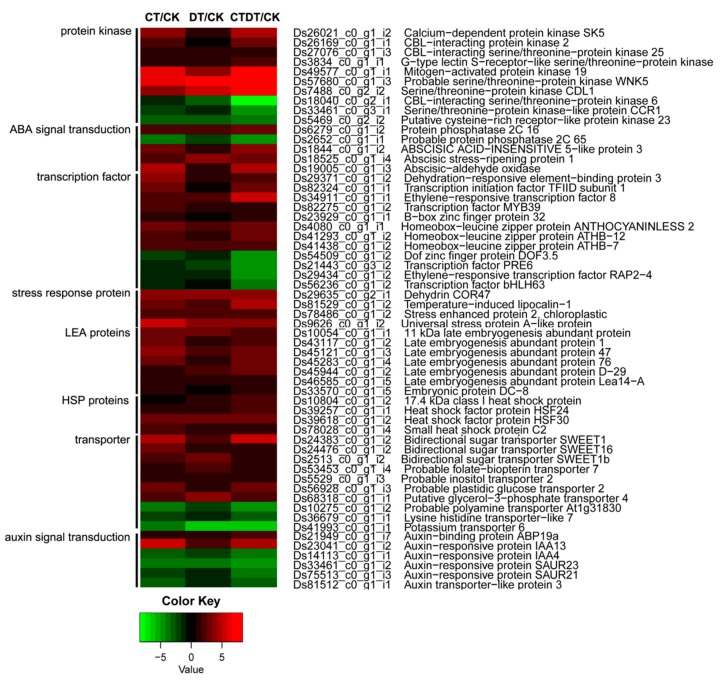
Expression profiles of 58 DEGs. The bar represents the scale of the expression levels of each gene (log_2_ RPKM) in the heat map. The red rectangles represent the up-regulation of genes, and the green rectangles represent down-regulation. All information for each gene can be found in Tables S1 and S2.

We produced a heat map based on the expression profiles to classify the key DEGs. The analysis showed that protein kinases and transcription factors constituted the largest number of DEGs (Figure 5). Among the 10 protein kinase genes, seven were up-regulated and three were down-regulated. The ten protein kinases included three CIPKs, two of which were up-regulated and the third of which was down-regulated. CIPKs are involved in the Ca^2+^-related signaling pathway that is central to the response of plants to both abiotic and biotic environmental stimuli [21,22,23]. Calcium is an important second messenger and it is thought that specific calcium ion signal patterns can result in the expression of targeted sets of genes [24]. CIPKs also interact with PP2C, which is involved in the ABA signal transduction pathway [25,26,27]. In the present study, two *PP*2*C* genes (*PP*2*C*16 and *PP*2*C*65) were identified: *PP*2*C*16 was up-regulated and *PP*2*C*65 was down-regulated. Among the 12 identified transcription factors, eight were up-regulated and the other four were down-regulated. Most transcription factors are associated with cold and drought stresses, such as DREB3 (dehydration-responsive element-binding protein 3), MYB39 (myeloblastosis 39), ERF79, and BBX32 (B-box zinc finger protein 32) [28,29,30,31,32]. Many stress response genes were identified, such as *COR*47 (cold-responsive gene 47), *LEAs* (late embryogenesis abundant) and *HSPs* (heat shock factor), and their expression was up-regulated. These genes are known to aid abiotic stress resistance in plants and help them cope with multiple stresses via ABA-dependent and ABA-independent pathways [33,34]. DREBs, also known as C-repeat binding factor (CBF), can regulate the expression of *COR* genes that confer freezing tolerance in plants [35]. The overexpression of *ZmDREB*2*A* results in the up-regulation of 44 genes, including *LEAs*, *HSPs*, and detoxification proteins, and increased drought tolerance in plants [36]. LEA proteins may have protective roles during cellular dehydration through physiological mechanisms such as hydration buffering, antioxidant protection and stabilization of sensitive processes [37,38]. As expected, the overexpression of *LEAs* from *Oryza sativa* enhances drought stress tolerance in transgenic rice [39,40]. In addition, ten transporter proteins and six auxin responsive proteins were identified. Most of the transporter proteins were up-regulated, whereas most of the auxin responsive proteins were down-regulated (Figure 5).

### 2.4. Validation of RNA-Seq Gene Expression

**Figure 6:**
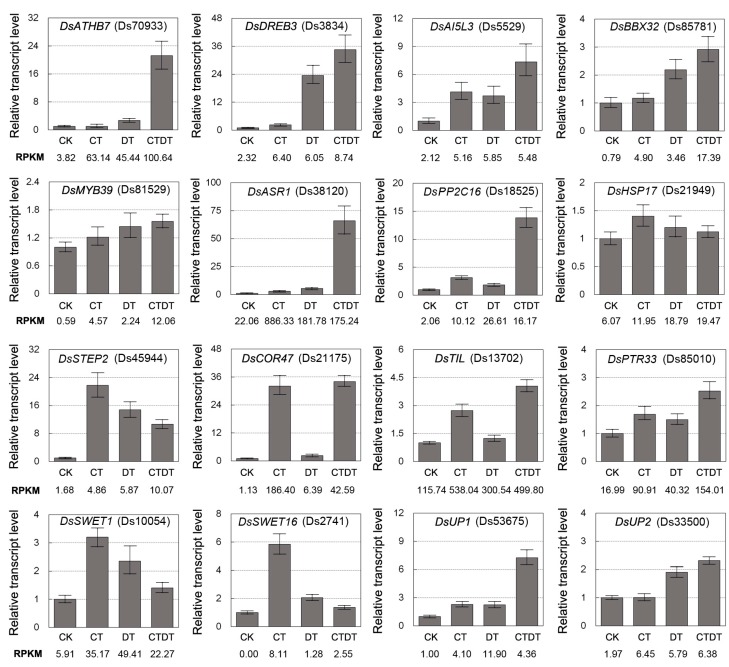
The relative expression levels of representative DEGs from CK, CT, DT, or CTDT samples. The *DsActin* gene was used as an internal control, and the transcript level in non-stressed seedlings was set as 1.0. Error bars represent SE (*n* = 3).

To confirm the reliability of the RNA-Seq data, quantitative real-time PCR (qRT-PCR) was performed on 16 randomly selected up-regulated genes. These DEGs included transcription factors (DsATHB7, DsDREB, DsAI5L3, DsBBX32, and DsMYB39), ABA signal-related proteins (DsASR1 and DsPP2C16), stress response proteins (DsHSP17, DsSTEP2, DsCOR47, and DsTIL), transporter proteins (DsPTR33, DsSWET1, and DsSWET16), and unknown proteins (DsUP1 and DsUP2). The 16 selected genes showed differential expression between control and stress-treated plants by qRT-PCR (Figure 6). The results of the qRT-PCR assay were correlated, with the RNA-Seq data (evaluated by RPKM), confirming the reproducibility of the RNA-Seq data.

Overall, we identified a large number of DEGs by transcriptome sequencing under cold and simulated drought stress, and annotated the regulatory pathways involving these DEGs. Our results provide further insights into the molecular mechanisms underlying the adaptation of *D. spiculifolius* to cold and drought conditions. However, there are limitations of the present study; for example, the simulation of drought by osmotic stress caused by mannitol could not completely replace the drought under natural conditions; the qPCR results of a few DEGs were not completely consistent with the RPKM data. Therefore, more reliable evidence from more experiments is desired for the validation of our results; for example, the increase in the type of stress and the experimental replications, qPCR validation at more time points, promoter analysis, examination of the vital physiological parameters, transgenic experiments, etc., are required and are being carried out for future reports.

## 3. Materials and Methods

### 3.1. Plant Growth Conditions and Stress Treatments

*D. spiculifolius* plants were grown in an open field at the Northeast Agricultural University (Harbin, China; 128.4° E, 45.0° N). *D. spiculifolius* seeds were surface sterilized and stratified at 4 °C for 2 days in the dark. After germination, the seedlings were grown on half-strength Murashige and Skoog (MS) medium (3% sucrose, 1% agar, pH 5.8) under a 12-h light/12-h dark photoperiod (100 μmol·m^−2^·s^−1^ light intensity) at 22 °C. Ten-day-old seedlings were assigned to control (no treatment, CK), cold (4 °C), simulated drought (300 mM mannitol), or combined cold and simulated drought (4 °C + 300 mM mannitol) treatment groups (10 seedlings per treatment) for 24 h. Three independent experiments were performed for each treatment condition. Seedlings from all the four groups were sampled simultaneously and were immediately stored at −80 °C until required for RNA extraction.

### 3.2. RNA-Seq

Total RNA from each sample was isolated using TRIzol reagent (Invitrogen, Carlsbad, CA, USA) according to the manufacturer’s instructions. The quality of the RNA samples was confirmed using a NanoDrop ND-8000 spectrophotometer (NanoDrop, Wilmington, DE, USA); the A260/A280 ratios of all of the samples ranged from 1.8 to 2.0. The integrity of the RNA samples was assessed with an Agilent 2100 Bioanalyzer; no sign of degradation was found. The RNA samples (mixed RNA of seedlings from three independent experiments according to 1:1 proportion) was sent to the Bionova Biotech (Beijing, China) for RNA sequencing. cDNA library construction and Illumina sequencing were performed according to the manufacturer’s instructions (Illumina, San Diego, CA, USA). Three technical replicates were performed per sample. The RNA-seq read data were deposited in the NCBI Sequence Read Archive (NCBI SRA) under the accession number SRX2637828.

### 3.3. Transcriptome Data Processing

The clean reads were obtained from the raw data by removing the adaptor sequences, reads with ambiguous bases “N”, low quality reads (*Q* value < 10), and fragments smaller than 20 bp in length. The de novo assembly of the *D. spiculifolius* transcriptome in the absence of a reference genome was accomplished using Trinity [41]. Trinity combines the reads with a certain length of overlap to form longer fragments, known as contigs. These contigs were subjected to sequence clustering to form longer sequences. Such sequences were defined as unigenes.

All of the assembled unigenes were compared with the public protein databases, including Swiss-Prot (http://www.expasy.ch/sprot), PFAM (Protein family, http://www.sanger.ac.uk/Software/ Pfam/), GO (Gene Ontology, http://www.geneontology.org), KOG (euKaryotic Ortholog Groups, http://www.ncbi.nlm.nih.gov/COG/), and KO (KEGG Ortholog, http://www.genome.jp/kegg/), using BLAST2GO analysis with a cut-off *E*-value of 10^−5^. After obtaining GO annotations for the assembled unigenes, WEGO software [42] was used to determine GO functional classifications for understanding the distribution of unigene functions. The unigenes were aligned to the COG database to predict and classify their possible functions. In the final step, KEGG pathway [43] annotations were performed to analyze the metabolic pathways and functions of the unigenes.

### 3.4. Screening of Differentially Expressed Genes (DEGs)

The expression levels of the unigenes were calculated using the RPKM (Reads per kilobase per Million reads) method [44]. The RPKM method eliminated the influence of the length and sequencing level of the gene. The formula used for determining the RPKM value was the following:

$$\mathrm{RPKM}=\frac{10^{6}C}{NL/10^{3}}$$

where *C* is the number of reads that uniquely aligned to one unigene; *N* is the total number of reads that uniquely aligned to all the unigenes; and *L* is the number of bases in the CDS of one unigene. All of the DEGs were mapped to each term of the GO and KEGG (Kyoto Encyclopedia of Genes and Genomes) databases, and significant pathways were defined based on a corrected *p*-value ≤ 0.05. The DEGs were screened with an false discovery rate (FDR) threshold of 0.05 or less and an absolute log_2_ ratio of 1 or more.

### 3.5. Heatmap and Protein–Protein Interaction (PPI) Analysis of DEGs

A heat map demonstrating the gene expression data was created using Java TreeView [45]. For the PPI analysis, the DEGs were used to retrieve the interacting genes with STRING 9.1 [46]. All the interactions in STRING were provided with a probabilistic confidence score (combined score). The PPI network was constructed using STRING and was visualized using Cytoscape. The proteins in the network served as nodes and the degree of a node was assessed as the number of interactions with other proteins. The proteins with high degrees were considered as the hub nodes.

### 3.6. Quantitative Real-Time PCR of DEGs

Sixteen DEGs were randomly selected from among the total of DEGs and were used to validate the reliability of the libraries. The primers for these assays (Table S3) were designed using Primer 5.0 software and quantitative real-time PCR (qRT-PCR) was carried out as described by Ren et al. [47]. The reference sequence was *DsActin*7 (Ds66780). Three biological and three technical replicates were performed per sample.

## 4. Conclusions

In this study, we provide the first report of the transcriptome data of *D. spiculifolius* under cold, simulated drought, or combined cold and simulated drought stresses using the Illumina HiSeq 2000 platform. A large number of DEGs involved in cold and drought stresses were identified. These DEGs and their functional annotations provide a valuable resource for genetic and genomic studies in *D. spiculifolius* plants. Functional characterization of the DEGs and their pathways needs to be performed, as they could potentially be used as targets for marker-assisted selection or transgenics to enhance stress tolerance.

## Figures and Tables

**Table 1:** Summary of Illumina transcriptome sequencing of *D. spiculifolius*.

Content	Control (CK)	Cold-Treated (CT)	Drought-Treated (DT)	Combined-Treated (CTDT)
Total reads	30,100,666	29,709,677	30,182,572	29,577,457
Total clean reads	23,624,696	22,495,628	23,317,141	22,965,345
Guanine and cytosine (GC) percentage	46.45	46.32	46.62	46.10
Total number of transcripts	184,782			
Total number of unigenes	112,015			
Mean length of unigenes (bp)	819			

**Table 2:** Number of functional annotations for all of the unigenes in public databases.

Annotated Database	Number of Unigenes	Percentage (%)
SwissProt	36,142	32.27%
Protein family (PFAM)	29,149	26.55%
Gene Ontology (GO)	29,739	26.54%
euKaryotic Ortholog Groups (KOG)	25,073	22.38%
KEGG Ortholog (KO)	10,845	9.68%

## Supplementary Materials

Supplementary materials can be found at www.mdpi.com/1422-0067/18/4/849/s1.
